# P-232. Reduction in Catheter-associated Urinary Tract Infections (CAUTIs) Post-Implementation of External Urethral Catheters (EUC) in the Intensive Care Units at a Quaternary Medical Center

**DOI:** 10.1093/ofid/ofae631.436

**Published:** 2025-01-29

**Authors:** George Bchech, Salman Bangash, Joseph Noonan, Arshpal Gill, Ryan Rothman, Charmaine Clarisse D Abalos, Adriana C Betancourth, Meilin Young, Nitin Bhanot

**Affiliations:** Allegheny General Hospital/Allegheny Health Network, PITTSBURGH, Pennsylvania; Allegheny Health Network, Pittsburgh, Pennsylvania; Golden Triangle, Baptist Health, Pittsburgh, Pennsylvania; Allegheny Health Network, Pittsburgh, Pennsylvania; Allegheny General Hospital/Allegheny Health Network, PITTSBURGH, Pennsylvania; Allegheny General Hospital, Pittsburgh, PA; Allegheny Health Network, Pittsburgh, Pennsylvania; Allegheny Health Network, Pittsburgh, Pennsylvania; Allegheny Health Network, Pittsburgh, Pennsylvania

## Abstract

**Background:**

Catheter associated urinary tract infections (CAUTIs) have significant clinical and financial impact on the US healthcare system. In a systematic review published in 2018, the attributable cost of a CAUTI was estimated to exceed $1000 (1). Indwelling urinary catheters increase risk for CAUTIs proportionate to the length of catheter use **(figure 1).** External Urethral Catheters (EUC) have the potential to reduce risk of CAUTIs. We conducted this quality improvement project to study the impact of EUC use on CAUTIs in the ICUs at our institution.

**Figure 1.**
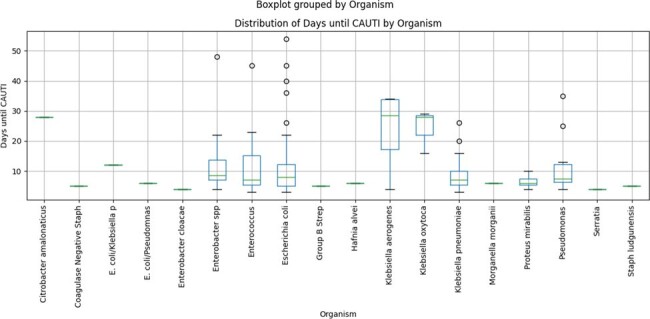
Length of Foley Catheter placement to CAUTI by organisms.

**Methods:**

EUC use was introduced in female (F) and male (M) patients since July 2018 and November 2021, respectively at our hospital. In this retrospective review, cases of CAUTIs were reviewed pre and post implementation of EUCs; in F from Jan 2015 to Jan 2021 and in M from Nov 2019 to Nov 2023 in patients admitted to the ICUs. National Healthcare Safety Network (NHSN) definition for CAUTIs was utilized for this project. Additionally, information on pathogens implicated in these infections was obtained.

**Figure 1:**
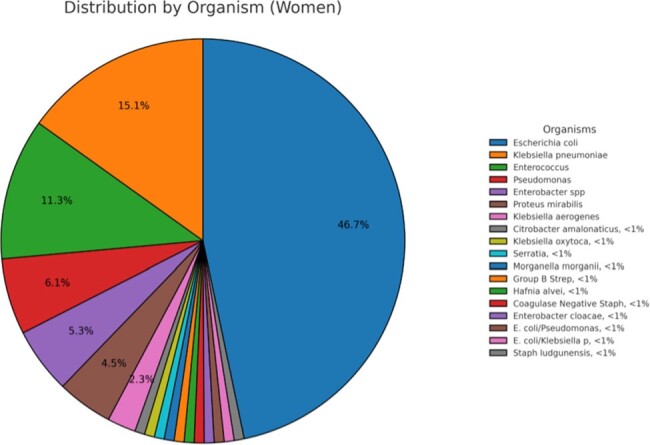
Distribution of organisms in Female population

**Results:**

There were a total of 157 CAUTIs noted in the study period; of the 132 F cases, 95 cases occurred pre and 37 cases post EUC implementation (RR 2.5 & RRR 61%). Of the 25 M cases, 15 cases were in pre and 10 cases post (RR 1.5 & RRR 33%) EUC use. Overall, there was an absolute risk reduction of 0.402. E.coli was the most common pathogen in both males (24%) and females (47%). There was an overall higher incidence of Enterococcus spp in the male population (20%) when compared to the female population (11.4%) **(Figure 2&3).**

**Figure 3:**
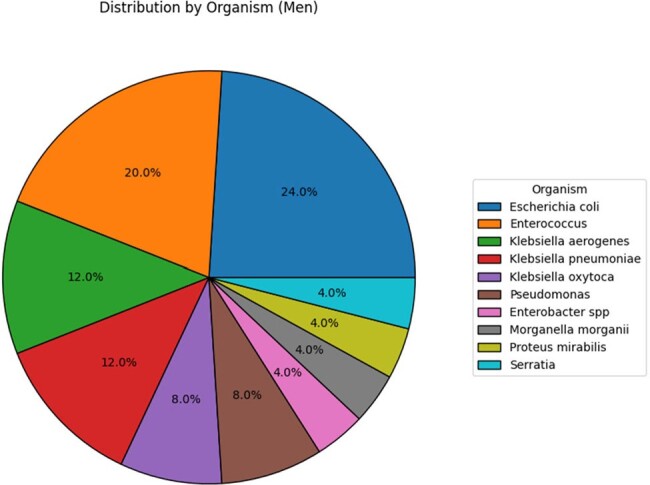
Distribution of organisms in male population

**Conclusion:**

Nosocomial infections are well described as a burden for ICU patients, but the results of this study demonstrate that there are possible interventions to mitigate CAUTIs. Clinicians and nursing staff should evaluate the need for indwelling urinary catheters on a daily basis. Favoring placement of EUC over indwelling urinary catheter has the potential to significantly reduce the burden of this disease.

**Figure:**
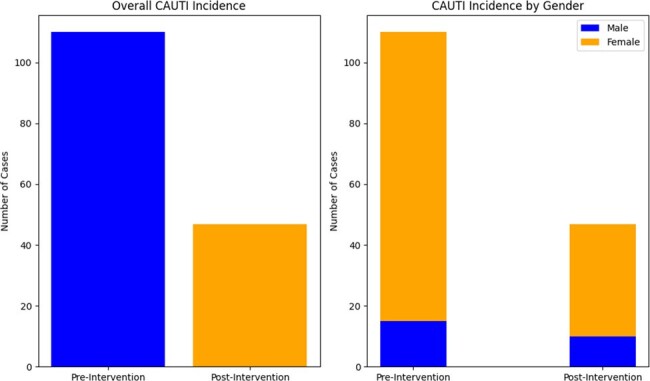

**Figure 4:** Total case pre and post implementation of EUC.

**Disclosures:**

**All Authors**: No reported disclosures

